# Mesoporous Polydopamine Nanoparticles Attenuate Morphine Tolerance in Neuropathic Pain Rats by Inhibition of Oxidative Stress and Restoration of the Endogenous Antioxidant System

**DOI:** 10.3390/antiox10020195

**Published:** 2021-01-29

**Authors:** Yaswanth Kuthati, Prabhakar Busa, Srikrishna Tummala, Vaikar Navakanth Rao, Venkata Naga Goutham Davuluri, Yen-Peng Ho, Chih-Shung Wong

**Affiliations:** 1 Department of Anesthesiology, Cathy General Hospital, Taipei 280, Taiwan; yaswanthk1987@gmail.com; 2 Department of Life Sciences, National Dong Hwa University, Hualien 97401, Taiwan; prabhakar.busa01@gmail.com; 3 Department of Chemistry, National Dong Hwa University, Hualien 97401, Taiwan; srkrishna011@gmail.com (S.T.); ypho@gms.ndhu.edu.tw (Y.-P.H.); 4 Institute of Biomedical Sciences, Academia Sinica, Taipei 11529, Taiwan; kanthuind89@gmail.com; 5 Department of Microbiology & Immunology, College of Medicine, National Cheng Kung University, Tainan 70101, Taiwan; goutibiotech76@gmail.com; 6 National Defense Medical Center, Institute of Medical Sciences, Taipei 280, Taiwan

**Keywords:** morphine antinociceptive tolerance, MPDA, neuropathic pain, morphine, reactive oxygen species, oxidative stress

## Abstract

Oxidative stress resulting from reactive oxygen species (ROS) is known to play a key role in numerous neurological disorders, including neuropathic pain. Morphine is one of the commonly used opioids for pain management. However, long-term administration of morphine results in morphine antinociceptive tolerance (MAT) through elevation of ROS and suppression of natural antioxidant defense mechanisms. Recently, mesoporous polydopamine (MPDA) nanoparticles (NPS) have been known to possess strong antioxidant properties. We speculated that morphine delivery through an antioxidant nanocarrier might be a reasonable strategy to alleviate MAT. MPDAs showed a high drug loading efficiency of ∼50%, which was much higher than conventional NPS. Spectral and in vitro studies suggest a superior ROS scavenging ability of NPS. Results from a rat neuropathic pain model demonstrate that MPDA-loaded morphine (MPDA@Mor) is efficient in minimizing MAT with prolonged analgesic effect and suppression of pro-inflammatory cytokines. Additionally, serum levels of liver enzymes and levels of endogenous antioxidants were measured in the liver. Treatment with free morphine resulted in elevated levels of liver enzymes and significantly lowered the activities of endogenous antioxidant enzymes in comparison with the control and MPDA@Mor-treated group. Histopathological examination of the liver revealed that MPDA@Mor can significantly reduce the hepatotoxic effects of morphine. Taken together, our current work will provide an important insight into the development of safe and effective nano-antioxidant platforms for neuropathic pain management.

## 1. Introduction

Neuropathic pain (NP) is often described as a chronic pain resulting from the damage or abnormal functioning of the nervous system. NP is a collection of diseases and conditions that affect the spinal cord, brain, and peripheral nervous system including diabetes, cancer, ischemic heart disease, alcoholism, and spinal cord injury [1]. In general, NP is resistant to over-the-counter pain relievers [2]. Opioids like morphine are often prescribed for the management of NP [3]. However, repeated administration of morphine can lead to morphine antinociceptive tolerance (MAT), which is characterized by a gradual loss of sensitivity to the anti-nociceptive effects, requiring an increase in dosage to achieve the desired level of analgesic effect which can aggravate the side effects and decrease the quality of life. Several studies have shown the role of oxidative stress in MAT [4,5]. Chronic morphine treatment is known to upregulate the genes of reactive oxygen species (ROS) along with simultaneous impairment of natural antioxidant enzymes leading to MAT [4,5]. Morphine is also known to cause cytotoxicity in cells at clinical concentrations [6].

Many studies have demonstrated that suppression of ROS by antioxidants or deletion of ROS causing genes such as nitric oxide synthase (NOS) could attenuate MAT in various animal models [4,7,8,9,10,11]. Though natural antioxidants have phenomenal ROS scavenging ability, clinical trials have shown limited success in preventing ROS related diseases with toxicity at high doses [12,13]. The clinical failure could be attributed to the following reasons: poor stability, low bioavailability, high renal clearance, the toxicity of metabolites, and short half-life. Recently, catalytic nanoparticles possessing antioxidant enzyme-like properties (nano-antioxidants) have gathered significant attention. Nano-antioxidants are easy to mass-produce and mimic multiple antioxidant enzymes with great stability and high bioavailability [14]. Among the nano-antioxidants studied, a variety of inorganic nanoparticles (NPS) including ceria, carbon, manganese, platinum, and selenium have shown remarkable efficacy in preventing ROS-related diseases [15]. However, cellular toxicity and clearance remain a major concern for the clinical transition of inorganic NPS [16].

Polydopamine (PDA) is a versatile organic biopolymer produced from the oxidation of dopamine. Due to its ease of synthesis and comparable chemical and physical properties to that of natural melanin, PDA has attracted considerable attention in the fields of biomedicine [17]. PDA has excellent biocompatibility and biodegradability in vivo without any cytotoxic effects [18,19]. Furthermore, its antibacterial, cell adhesion and cell proliferation properties make it an ideal material for surface coating of implants and other inorganic nanocarriers used for biological applications [20,21]. Moreover, PDA-NPS alone have shown excellent antioxidant properties in vivo by scavenging ROS, preventing the brain injury from ischemia, as well as healing acute lung injury and alleviating periodontal inflammation without side effects [22,23,24]. However, the limited surface area of PDA-NPS limits their application in loading pharmacological drugs. Considering this, porous NPS with a mesoporous structure were proven to have several advantages over non-porous equivalents with desirable properties such as ease of surface functionalization, high surface area, and high drug payload [25]. Despite a few innovative works on the application of mesoporous polydopamine (MPDA-)NPS for anti-cancer drug delivery and cancer diagnosis, the efficacy of MPDA-NPS for analgesic drug delivery has not yet been demonstrated to our best knowledge [26,27,28]. Nevertheless, several nanoparticle-based analgesic drug delivery systems have shown a promising efficacy in preclinical studies [29]. Encapsulation of analgesics into nanomaterials like liposomes, chitosan and poly(lactic-co-glycolic acid) have shown sustained analgesic release with prolonged analgesic effects and lower cytotoxic effects [30].

In our current work, MPDA-NPS were developed for the delivery of opioid analgesic morphine to treat neuropathic pain. At the in vitro level, MPDA-NPS displayed excellent 2,2-diphenyl-1-picrylhydrazyl (DPPH) radical scavenging ability and significantly suppressed the elevation of morphine induced intra-cellular ROS levels. Furthermore, using a partial sciatic nerve transection (PSNT) model of neuropathic pain in rats, we demonstrated that delivery of morphine with MPDA significantly delayed MAT by maintaining redox balance through the restoration of endogenous antioxidant enzymes in the liver along with the suppression of microglial cell activity in the spinal dorsal horn. Moreover, delivery of morphine with MPDA had significant hepatoprotective effects against morphine induced hepatotoxicity through the suppression of liver enzymes. Taken together, our current work provides a promising platform for designing efficient biocompatible nano-antioxidants for applications in the field of biomedicine.

## 2. Materials and Methods

### 2.1. Reagents

Dopamine hydrochloride was purchased from Carbosynth (Berkshire, UK). Trimethyl benzene was purchased from Alfa Aesar (Tewksbury, MA, USA). Morphine, Pluronic F127 (F127), and Tris were purchased from Sigma-Aldrich (St. Louis, MO, USA). DPPH (2,2-Diphenyl-1-picrylhydrazyl) was purchased from Med chem express (Princeton, NJ, USA).

### 2.2. Animals

The methods used in our research were evaluated and approved by the Animal Care and Use Committee of the National Defense Medical Center, Taipei, Taiwan and fulfill the regulations specified by the National Institute of Health Guide for the Care and Use of Laboratory Animals. (IACUC109-018). The 7-week-old male Wistar rats were obtained from BioLASCO Taiwan Co., Ltd., Taiwan and housed with soft bedding material on a 12-h night/day cycle with free access to food and water.

### 2.3. Characterizations

Transmission electron microscopy (TEM) images were taken with Tecnai G2 TF 20 Super Twin electron microscope and the images were obtained through Digital Micrograph (Gatan, Inc., Pleasanton, CA, USA). The zeta potential charge and particle size distribution of the NPS were measured at physiologic pH through dynamic light scattering (DLS) using a Malvern-Zetasizer Nano ZS 90 (Malvern, Worcestershire, UK). UV-vis spectra were analyzed using a Genequant-1300 series spectrophotometer (Biochrom, Holliston, MA, USA). For confirmation of surface functional groups, FT-IR spectra were recorded on ALPHA spectrometer (Bruker Optics Inc., Billerica, MA, USA) by mixing the samples with dried potassium bromide (KBr) pellets. The porous characteristics of the nanomaterial before and after drug loading were studied using N2 adsorption–desorption isotherms at 77 K on a Micrometric ASAP 2020 apparatus (Micromeritics, Norcross, GA, USA). The crystalline structure of the materials was examined using D2 PHASER X-ray diffractometer (Bruker, Karlsruhe, Germany. The physical characteristics and drug loading were determined by Thermogravimetric analysis of a TGA-DTA curve on TGA Q50 V20, 13 Build 39 (TA instruments, New Castle, DE, USA). The temperature was gradually increased from ambient to 600 °C at a 20 °C/min rate under dry 616 nitrogen purge at a flow rate of 20 mL/min.

### 2.4. Synthesis of MPDA

The NPS were synthesized by following the one-pot synthesis method reported by Chen et al. [31]. At first, 1.44 g of F127 and 1.44 g of trimetyl benzene (TMB) were solubilized in a mixture of ethanol (240 mL) and water (260 mL). After stirring for half an hour, a cocktail solution of Tris (360 mg) dissolved in 40 mL double distilled water was added to the mixture. After five minutes, 360 mg dopamine hydrochloride was added, and the contents were stirred for 24 h at room temperature and collected by centrifugation. The NPS were thoroughly rinsed with acetone and ethanol twice. Finally, the templates were removed through sonication using a mixture of ethanol and acetone (2:1 *v*/*v*) thrice. The product was named as MPDA-Extd.

### 2.5. Morphine Loading

Template extracted MPDA-NPS were added to phosphate buffered saline (PBS) solution containing morphine at a mass ratio of drug to MPDA equal to 2:1 and the contents were stirred overnight at room temperature. The morphine loaded MPDA-NPS were collected by centrifugation, and then washed several times with water and PBS to remove free morphine and finally the obtained MPDA-loaded morphine (MPDA@Mor) NPS were stored in 99.9% ethanol for further use. The product is denoted as MPDA@Mor. The drug loading was calculated by the following formula.

Drug loading percent (%) = (weight of the drug in NPS/weight of drug fed initially) × 100.

### 2.6. In Vitro Release Study

In vitro release experiments were performed in a normal saline solution. The experimental conditions were as follows: 5 mg of MPDA@Mor samples were added into 1 mL of buffer and maintained at 37 °C by placing samples on a shaking apparatus 150 rpm, and then the particles were centrifuged (14,000 rpm for 20 min) at different time intervals (0–10 h). The percentage of drug release was determined by Uv-visible spectral measurements at 285 nm.

### 2.7. DPPH Radical Scavenging Assay

The DPPH radical scavenging activity of MPDA and MPDA@Mor were determined by using the procedures reported earlier [32]. NPS at different concentrations (0–50 μg/mL) were prepared using serial dilution in ethanol. A suspension of 0.1 mL of different NPS were added to 1 mL of 0.1 mM newly prepared DPPH solution in ethanol. The mixtures were repeatedly agitated for 10 min and the NPS were separated using centrifugation and the absorbance of supernatants was recorded at 517 nm in a UV-vis spectrophotometer. The radical scavenging efficiency was defined as a measure of the decrease in absorbance intensity by using the following formula.

DPPH scavenging activity (%) = [(Abs _control_ − Abs _sample_)/(Abs _control_)] × 100

### 2.8. LDH Assay

NPS toxicity was assessed by Roche lactate dehydrogenase (LDH) cytotoxicity detection kit (Sigma-Aldrich inc, St. Louis, MO, USA). This test quantifies the leakage of cytoplasmic enzyme LDH from the cells upon cell membrane damage. CNS-1 cells obtained from ratus norvegicus were purchased from American type culture collection (ATCC) and 1 × 10^4^ cells were cultured in 96-well plates using 10% Dulbecco’s modified Eagle’s medium (DMEM) for a day and treated with increasing concentrations of NPS (10, 25, 50, 100, and 150 μg/mL). After 48 h of incubation, supernatants were collected and incubated with the reaction mixture. The catalyzed conversion of LDH results in the conversion of tetrazolium salt to Formosan, which can be quantified at 490 nm. TitonX-100 served as a positive control and DMEM as a negative control.

### 2.9. ROS Evaluation in Cells

The human monocytic cells (THP-1) derived from acute monocytic leukemia patients were obtained from thermofischer and were either untreated (DMEM) or treated with either morphine (5 μg/mL), MPDA (10 μg μg/mL) or MPDA@Mor (10 μg/mL) and incubated for 12 h. After incubation 10 µM 2′,7′-dichlorodihydrofluorescein diacetate (DCFDA) molecular probe indicator was added and performed 30 min post-incubation. Finally, the readings were recorded at the FL-1 channel using a flow cytometer. ROS generation was measured in triplicate. The data shown are the mean ± SD values of 3 individual experiments.

### 2.10. Establishment of Neuropathic Pain

Partial sciatic nerve transection surgery was performed by following the procedure reported previously [33,34]. The sciatic nerve from the left leg was carefully pulled until mid-thigh level, then a prolene 7-0 needle was introduced into the nerve just cranially to the branch stretching to the musculus biceps femoris, half of the nerve was transacted in a ventrocranial way up to the ligature. In the sham rats, the sciatic nerve was exposed, and the surgical site was closed with sutures. From the surgery day to day 7 the paw withdrawal thresholds were determined in sham-operated rats and PSNT rats to mechanical stimuli. Rats showing any sign of motor deficit were eliminated.

### 2.11. Evaluation of Tolerance Induction

The rats were made tolerant to morphine using the method reported by a previous study [35]. The groups of rats received saline or morphine (15 mg/kg) or MPDA@Mor (15 mg morphine equivalent weight) NPS once a day in the morning for 13 days intraperitoneally (i.p) from day 9 post PSNT. One-hour post-injection the paw withdrawal thresholds are measured to find the post-drug threshold for each rat to confirm the development of MAT.

### 2.12. Behavior Test for Tactile Allodynia

The paw sensitivity was measured in the left hind paw of rats using an automatic Dynamic Plantar Aesthesiometer. Rats were placed in separate open see-through plastic containers (25 cm long × 10 cm wide × 14 cm high) with a base containing metal-mesh. The rats were initially acclimatized for 20 min before each testing session. The paw withdrawal threshold was determined by progressively increasing the strength from 1 to 50 g in 1-g steps using a tiny blunt-end metal rod (0.5 mm) pointed towards the mid-plantar region of the paw. The hind paw reflex time was recorded three times for each animal at 2-min time intervals and the average was calculated with 50 g as a cut-off threshold to avoid paw damage.

### 2.13. Maximal Possible Effect Determination

After the single injection of morphine or MPDA@Mor, the paw withdrawal threshold (s) is expressed as the percentage of maximal possible effect (%MPE) using the equation below:

%MPE = (Post-drug threshold (s)) − (Baseline paw withdrawal threshold on day 7 after sciatic nerve transection (s))/(Cut-off value (s)) − (Baseline paw withdrawal threshold on day 7 after sciatic nerve transection (s)) × 100.

### 2.14. Spinal Cord Preparation and Western Blotting Analysis

After behavioral tests, rats were euthanized under anesthesia with isoflurane (Abbott Laboratories Ltd., Queenborough, Kent, UK), and the left dorsal quadrant section of the lumbar spinal cord is isolated and preserved at −80 °C. The tissue sections were homogenized in ice-cold 1X radioimmunoprecipitation assay lysis buffer using a cell disruptor under sonication (Misonix, Inc. Newtown, CT, USA), then the contents were centrifuged at 13,000 RPM for half an hour at 4 °C. The supernatant portion is carefully collected, quantified using Bradford protein assay. The protein denaturing is done by heating at 90 °C for 10 min in an equal volume of reducing sample buffer and separated using 12% SDS-PAGE and proteins were transferred on to a polyvinylidene fluoride membrane (Pall, Ann Arbor, MI, USA) and kept for blocking using 5% skimmed milk in tris-buffered saline (0.05% Tween 20 in tris-buffered saline). Primary antibodies against CD-11B antibody (Genetex Cat No. GTX134493, Alton Pkwy Irvine, CA, USA) were incubated at 4 °C overnight followed by washing with TBST. Furthermore, they were incubated with horseradish peroxidase conjugated goat anti-rabbit antibody (Leadgene Biomedical Cat# 20202, Tainan, Taiwan) for another 3 h and detected using an Enhanced Chemiluminescence Western Blotting Kit (Advansta, Menlo Park, CA, USA).

### 2.15. Pharmacokinetic Studies

The pharmacokinetic studies were done using the previously reported protocols with minor changes [36]. Rats received one intraperitoneal (i.p.) injection of morphine or MPDA@Mor. The blood samples were collected from the tail vein using a 2% isoflurane mask at different time points (5 min, 0.5, 2, 4, 6, 8, 12, and 24 h) after drug administration. The heparinized blood samples were centrifuged, and plasma was frozen and stored at -80 °C. The samples were purified through the liquid–solid extraction method reported by Krugner et al. [37]. After purification, the samples were injected into a gas chromatography system (Agilent Technologies 7890B, Santa Clara, CA, USA) armed with a mass selective detector (Agilent Technologies 5977A MSD, Santa Clara, CA, USA) and an autoinjector Gerstel MPS 2XL. Totals of 429 and 432 ions (specific for morphine) were chosen for quantification of the drug.

### 2.16. Measurement of TNF-α and NFkb

The levels of tumor necrosis factor- α (TNF-α) and nuclear factor kappa light chain enhancer of activated B (NF-kB) cells from dorsal root ganglion (DRG) supernatants were measured using commercial enzyme-linked immunosorbent assay (ELISA) kits (YL Biont, Shanghai, China). Briefly, samples were added to the ELISA plate and were incubated for 1 h at 37 °C. After washing the unbounded contents, plates were incubated with stains and additionally incubated for about 20 min at 37 °C. Finally, a stop solution was added, and plates were read at 450 nm. Standard curves were plotted to determine the value of samples.

### 2.17. Measurement of Endogenous Antioxidants and Malondialdehyde (MDA)

The livers of the rats were removed, weighed, and washed in normal saline (0.9%) buffer and stored in liquid nitrogen. The samples were homogenized, and the pellets were obtained by centrifuging the contents at 12,000 g and the supernatants were used for enzyme assays. Whole homogenates were used for quantification of lipid peroxidation whereas a small sample of supernatant was used for superoxide dismutase (SOD) and catalase (CAT) estimation. SOD activity was determined following the method reported by Marklund et al., by exploiting inhibition of pyrogallol autoxidation at pH 8 [38]. CAT activity was determined by H_2_O_2_ consumption, following the method adopted by Pieper et al. [39]. In brief, ethanol was added (1:100 *v*/*v*) to the supernatants and incubated for half an hour in an ice bath followed by the addition of 1% Triton X-10 and incubation for 15 min. An amount of 500 μL of this suspension was placed into a glass cuvette and 250 μL of 30 mM H_2_O_2_ was added. After 15 s the absorbance was recorded at 240 nm. The lipid peroxidation was determined by following the thiobarbituric acid reactive substance (TBARS) assay reported by Gene et al. [40]. The formation of MDA was measured spectrophotometrically at 532 nm.

### 2.18. Serum Biochemical Analysis

For determination of liver alanine aminotransferase (ALT), aspartate transaminase (AST), and LDH enzyme levels in serum, blood was collected from the tail vein and serum was isolated by centrifugation at 3000 rpm for 10 min at 4 °C and frozen at −20 °C. The serum levels of the activity of ALT, AST, and LDH were measured through a Hitachi, Type 7170 automated biochemical analyzer (Diamond diagnostics, Budapest, Hungary).

### 2.19. Hematoxylin and Eosin Staining

After behavioral tests, the histological analysis was performed by examining the morphological changes induced by morphine in the rat livers. Rat livers were embedded in the TissueTek optimal cutting temperature (OCT) compound (Sakura Finetek, Torrance, CA, USA). The OCT sections were cut (5 μm), mounted on Superfrost Plus slides (Thermo Fisher Scientific, Rockford, IL, USA), dried for an hour at room temperature, stained for analysis using a hematoxylin and eosin (H and E) staining kit (Abcam, ab245880, Cambridge, MA, USA). The slides were checked under a fluorescent microscope and images were captured.

### 2.20. Statistical Analysis

Unpaired data were evaluated using Student’s t-test to compare two mean values, and one-way analysis of variance (ANOVA) followed by Tukey’s multiple-comparison test which was used to compare more than two mean values. All data were expressed as mean ± standard deviation. In all analyses, a *p* ≤ 0.001 (***), *p* ≤ 0.01 (**), and *p* ≤ 0.05 (*) was considered statistically significant.

## 3. Results and Discussion

**Figure 1:**
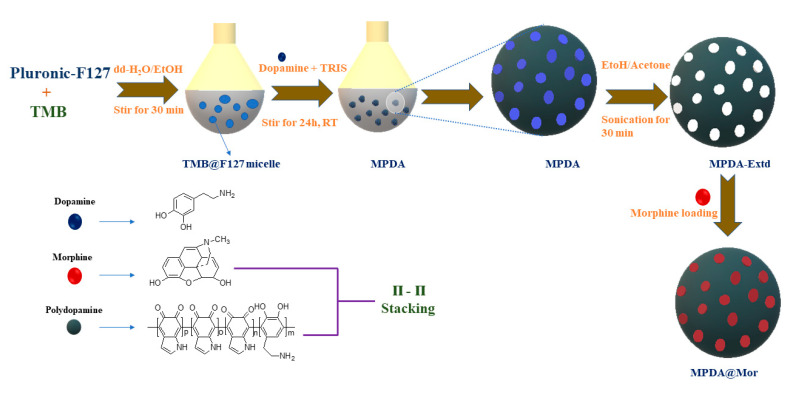
A schematic illustration for the synthesis and drug loading of mesoporous polydopamine (MPDA), and morphine drug loading in to MPDA nano-antioxidants.

MPDA-NPS were synthesized by emulsion-induced interface assembly using F127 and TMB (Figure 1) according to the procedure described by Chen et al. [31].

**Figure 2:**
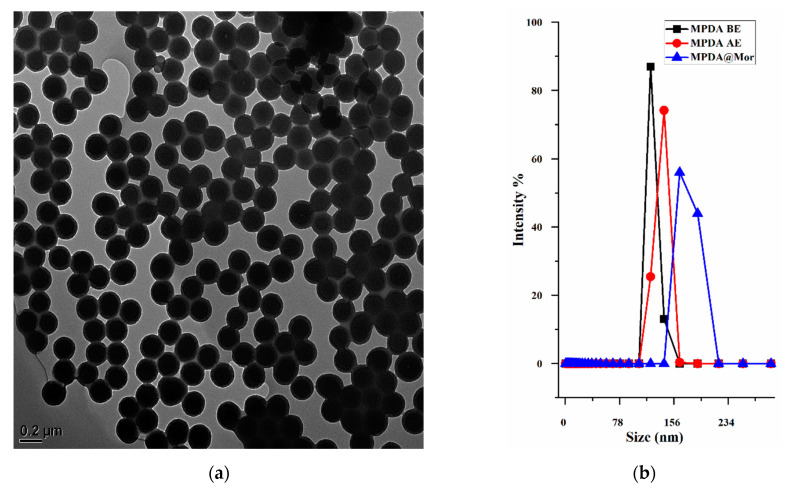

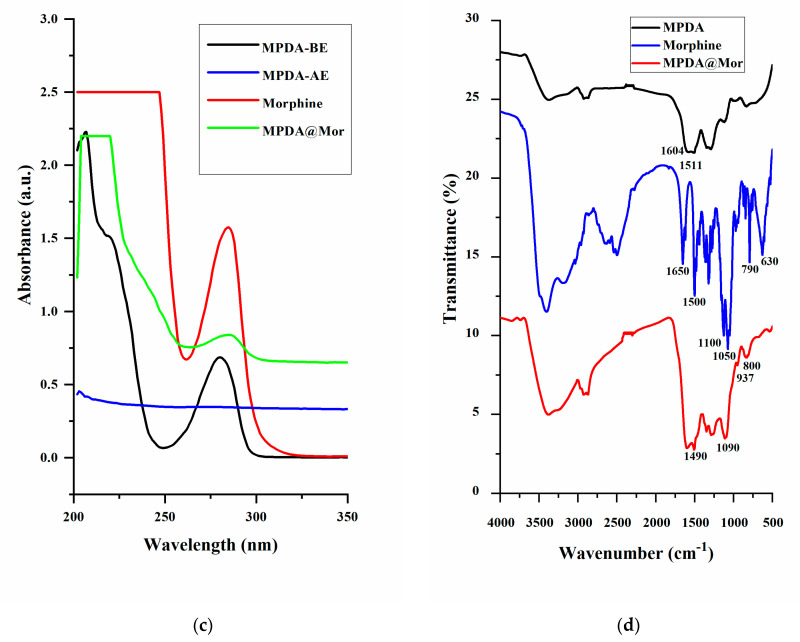
(**a**) TEM image of template extracted MPDA (**b**) hydrodynamic diameter distribution of BE and AE MPDA and MPDA loaded morphine (MPDA@Mor). (**c**) UV-vis and (**d**) FT-IR spectra of morphine, MPDA and MPDA@Mor.

Template extracted MPDA-NPS displayed spherical and even particle size distribution with a diameter of 150–200 nm (Figure 2a). The hydrodynamic size of different MPDA samples is determined by dynamic light scattering (DLS) (Figure 2a). As-synthesized MPDA nanoparticles displayed slightly smaller size (141.71) than the template extracted MPDA (149.63). This is presumably due to the existence of a hydrate layer over template extracted MPDA due to the repeated washing with the aqueous solution, thereby causing a slightly larger hydrodynamic diameter. The DLS size of template extracted MPDA is like the size observed under TEM with a low polydispersity index (PDI) (Figure 2b). However, the adsorption of morphine resulted in a significant increase in NP size from 149.63 nm to 190.13 nm, indicating successful binding of morphine to MPDA (Table 1). To further verify the loading of morphine, the zeta-potential of different MPDA samples was measured in water (Table 1).

The zeta-potential of as-synthesized MPDA showed a slight negative charge (−20 mv) and removal of template slightly increased the negative charge to −32.09 mv and morphine loading further decreased the negative charge to −14.01 mv. The negative charge in MPDA is ascribed to the presence of hydroxyl groups in the MPDA framework which imparts hydrophilic characteristics. Furthermore, the presence of amine and phenolic functional groups impart zwitterionic properties at isoelectric pH (pH 4–4.5). However, at physiologic pH (pH 7.2–7.4), the deprotonation of phenolic groups results in a negative surface charge [41]. The slight reduction in zeta potential after morphine loading could be attributed to the interactions between amine groups in morphine with hydroxyl groups in MPDA through π–π stacking.

MPDA and MPDA@Mor NPS were further characterized by ultraviolet–visible (UV-vis) and Fourier Transform Infrared (FTIR) spectroscopy. UV-vis spectra of ethanolic solutions of MPDA, morphine, and MPDA@Mor were obtained to study the spectral characteristics of the samples (Figure 2c). Before template extraction, MPDA showed a relative dopamine absorption peak at 280 nm, which can be attributed to the substantial amount of dopamine (Figure 2c black curve). Template extraction resulted in the loss of 280 nm peak (Figure 2c blue curve), which can be attributed to the decline in dopamine levels. Similar spectral characteristics were observed in previously reported studies [42,43]. Free morphine showed a characteristic absorption peak of morphine at 285 nm (Figure 2c red curve). UV-vis spectra of MPDA@Mor showed typical absorption peak of morphine at 287 nm, with a slight shift and broadening indicating successful morphine loading in to MPDA (Figure 2c green curve). To investigate the functional groups of the samples FTIR spectral characteristics of MPDA, morphine, and MPDA@Mor NPS are performed (Figure 2d). MPDA samples show peaks at 1511 and 1604 cm^−1^ resulting from the indole groups in MPDA [44]. The broad peak ranging from 3200 to 3500 cm^−1^ is ascribed to the hydroxyl groups and water [44]. For morphine, the peaks ranging from 1600 to 1650 cm^−1^ and 630 to 650 cm^−1^ correspond to C–C stretching vibrations and deformation vibrations and are also found in other opioids in the same range [45]. The strong signals at 1050 cm^−1^ are from C–O–C stretching vibrations [45]. The band at 970 and 1100 corresponds to the C=O stretching vibration of morphine [45]. The distinguishing morphine bands in MPDA@Mor at 1490, 1090, 937 and 800 cm^−1^ confirm the presence of morphine.

**Figure 3:**
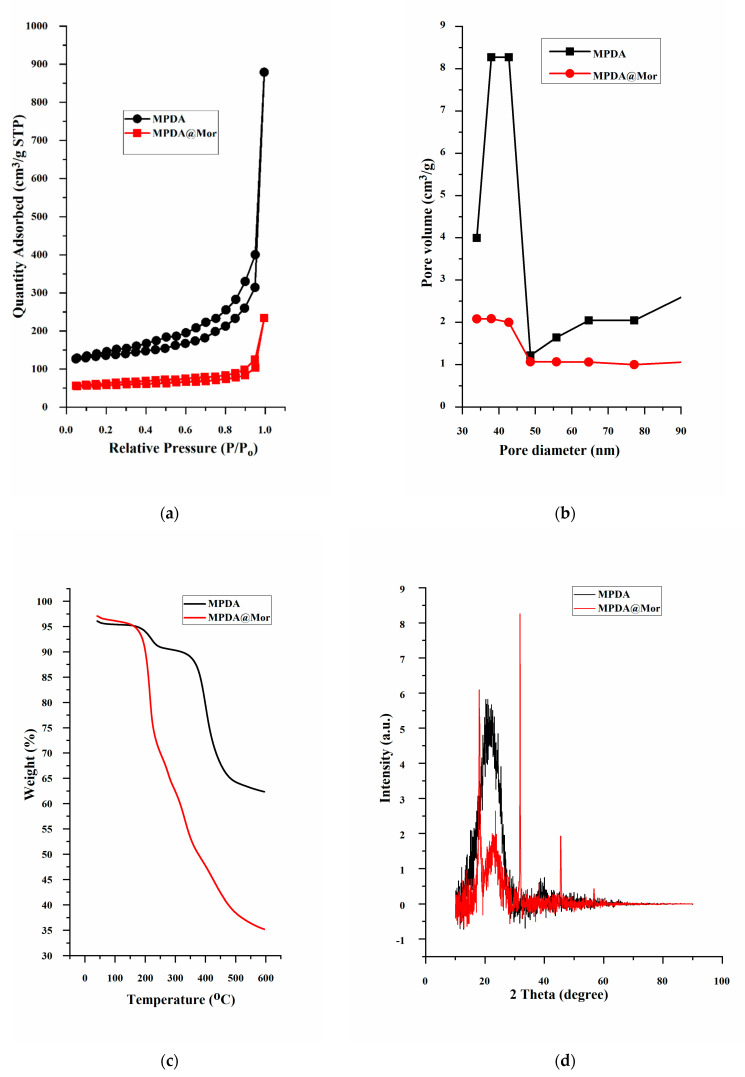
(**a**) Nitrogen adsorption–desorption isotherms, (**b**) pore size distribution, (**c**) thermogravimetric analysis and (**d**) powder X-ray diffraction patterns of MPDA and MPDA@Mor.

The mesoporous characteristics of the MPDA-NPS and changes attained after successive morphine loading are verified through nitrogen adsorption-desorption isotherms experiment by using Brunauer–Emmett–Teller (BET). MPDA-NPS exhibited a type IV isotherm with a BET specific surface area of 27.906 m^2^g^−1^ with a total pore volume of 0.282 cm^3^ g^−1^, with a mesopore size of about 33.939 nm (Figure 3a,b). After adsorption of morphine, MPDA@Mor samples displayed a reduced BET surface area of 15.392 m^2^g^−1^ and a pore volume of 0.277 cm^3^ g^−1^ and a mesopore size of about 30.264 nm due to adsorption of morphine into the mesopores. Thermal degradation curves of MPDA and MPDA@Mor were analyzed by heating samples in an N_2_ atmosphere up to 600 °C (Figure 3). Both MPDA and MPDA@Mor samples have around 5% weight loss at ≈100 °C which can be attributed to the loss of absorbed moisture. The MPDA samples have a weight loss starting at around 300 °C which is in good agreement with the thermal decomposition of PDA homopolymers as reported previously [46]. The residual mass of MPDA is ∼60% (Figure 3c black curve). The TGA curve of MPDA@Mor shows ∼65% weight loss at 200–600 °C resultant from morphine degradation (Figure 3c red curve) [47]. The appearance of weight loss at two different stages of thermal degradation confirms the interaction between MPDA and morphine. The residual weight loss for MPDA@Mor was almost 35%. This is in line with other reports that studied the thermal degradation properties of morphine [47].

Previously, a few studies have used MPDA-NPS for anticancer drug loading through π−π stacking [26]. To verify the existence of such π−π stacking in MPDA, we performed powder X-ray diffraction (PXRD) on MPDA and MPDA@Mor. The MPDA sample shows a broad peak with a 2θ of 23.4° which can be attributed to the mesoporous characteristics of the MPDA nanomaterials (Figure 2d) [48]. The materials showed a d-spacing of approximately 3.8 Å which is consistent with previously reported π-stacked structures which can enable charge transfer properties between MPDA monomers [49]. In MPDA@Mor samples, there are characteristic peaks of morphine and other opioid drugs with a 2θ of 16.4° and they are consistent with those calculated from the structures stated in Cambridge Structural Database (CSD), reported before [50]. All these data suggested the existence of π−π interaction between MPDA and morphine.

**Figure 4:**
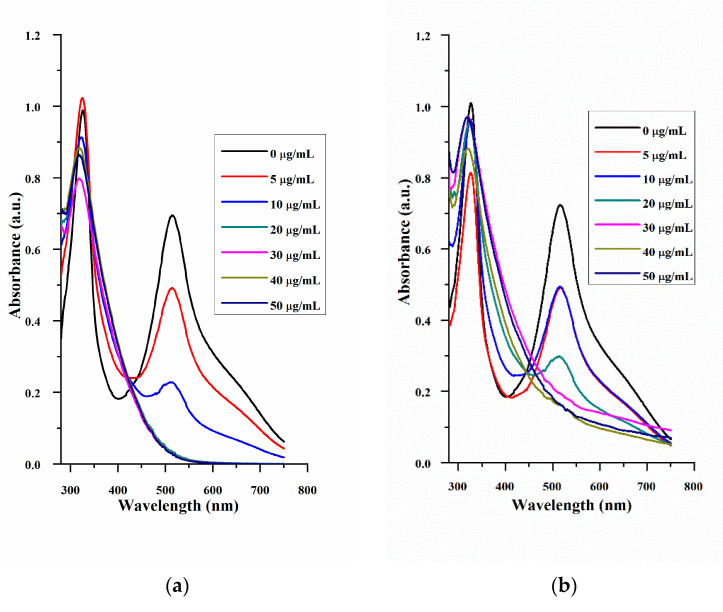

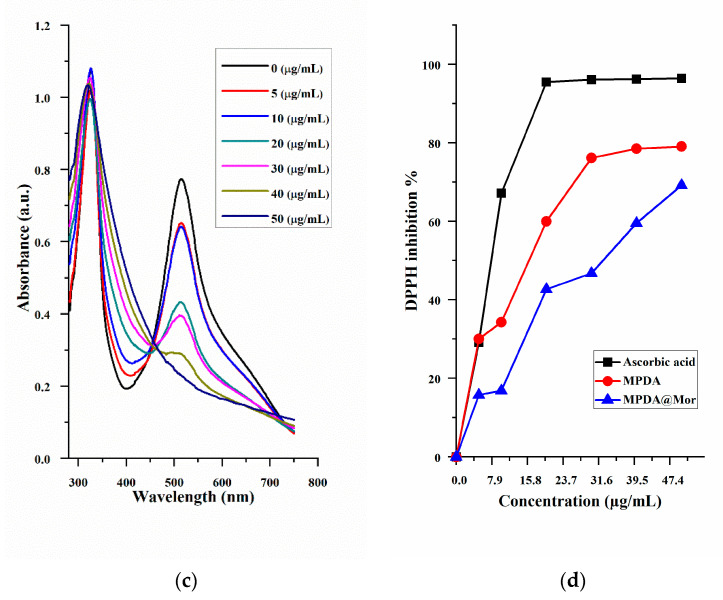
UV-vis absorption spectra signifying the DPPH free radical scavenging efficacy of (**a**) ascorbic acid. (**b**) MPDA and (**c**) MPDA@Mor. (**d**) DPPH free radical scavenging percentage at different concentrations of ascorbic acid, MPDA, and MPDA@Mor.

The antioxidant properties of MPDA and MPDA@Mor were estimated by using the DPPH free radical scavenging method. Ascorbic acid is used as a positive control (Figure 4). DPPH samples showed a strong peak at 517 nm (Figure 4). The antioxidant properties increased with the increase in the concentration of ascorbic acid and NPS. The radical scavenging activity for MPDA at 30 µg/mL was estimated to be around 80% (Figure 4d, red curve) which is close to 92% acquired for the standard ascorbic acid at the equivalent concentration (Figure 4d, black curve).

**Figure 5:**
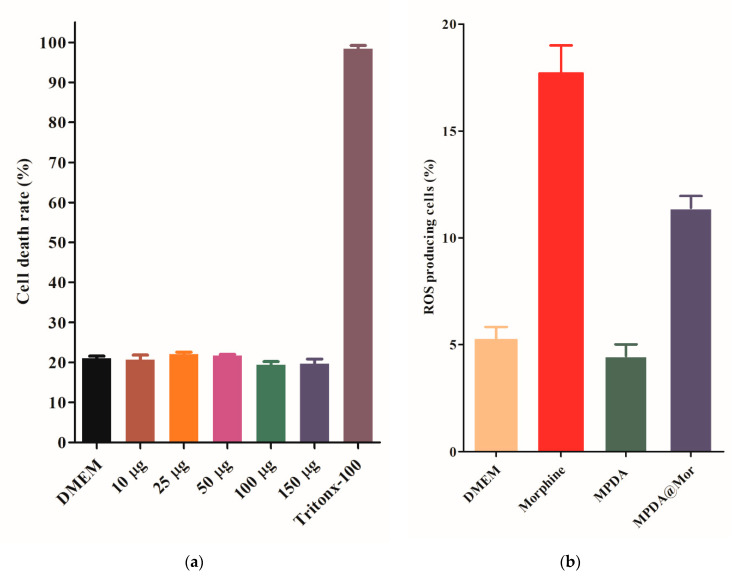
(**a**) Effect of MPDA on lactate dehydrogenase (LDH) leakage in CNS-1 neuronal cells. Cytotoxicity was determined by quantification of LDH release after 24 h of exposure to MPDA- nanoparticles (NPS) (10–150 μg/mL). Data are means ± SD. Results were calculated as the percentage of the positive control (Triton X-100-lysed cells) (n = 3). (**b**) Determination of cellular reactive oxygen species (ROS) by DCFDA assay in THP cells.

The IC_50_ value for the MPDA was found to be around 16 µg/mL whereas MPDA@Mor had an IC_50_ value of 32 µg/mL, showing that MPDA@Mor could still act as a strong antioxidant (Figure 4d, red and blue curves). The 50% reduction in IC_50_ value in MPDA@Mor could be attributed to 50% morphine loading in the samples. LDH assay was performed to examine the cytotoxic effects of MPDA-NPS in the CNS-1 tumor cell line (Figure 5a). LDH assay quantifies the amount of LDH leakage upon cell damage. The tumor cell line is chosen for the cytotoxic evaluation of several NPS, as tumor cell lines are known to possess a higher concentration of LDH [51]. Treatment with increasing concentrations of MPDA did not increase LDH leakage compared to the negative control DMEM, whereas treatment with positive control triton × 100 resulted in 100 percent lysis. At all the tested concentrations (10–150 µg/mL), the NPS showed exceptional biocompatibility.

Repeated administration of opioids including morphine is known to damage cells and tissues through the generation of ROS and suppression of antioxidant genes resulting in opioid-induced disorders such as addiction and MAT [4,52,53]. The supplementation of antioxidants is shown to improve the disturbance of redox homeostasis induced by morphine [7,8,9,10]. To verify this phenomenon THP cells were treated either with MPDA, morphine, and MPDA@Mor with DMEM as a positive control. The treatment with morphine significantly increased the ROS levels (Figure 5b, red bar) compared to all groups. MPDA@Mor samples efficiently reduced the ROS up-regulation indicating that the morphine induced ROS scavenging capabilities thereby maintaining a redox-environment (Figure 5b, violet bar). Our results show that exogenous antioxidant properties of MPDA can abate the morphine induced ROS in cells.

**Figure 6:**
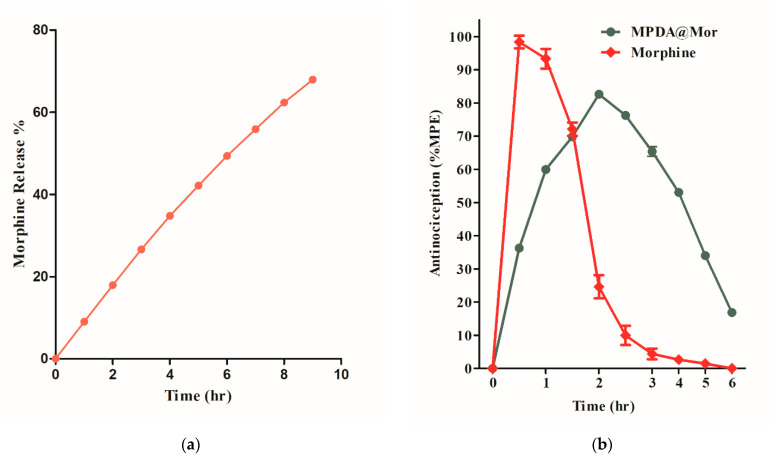

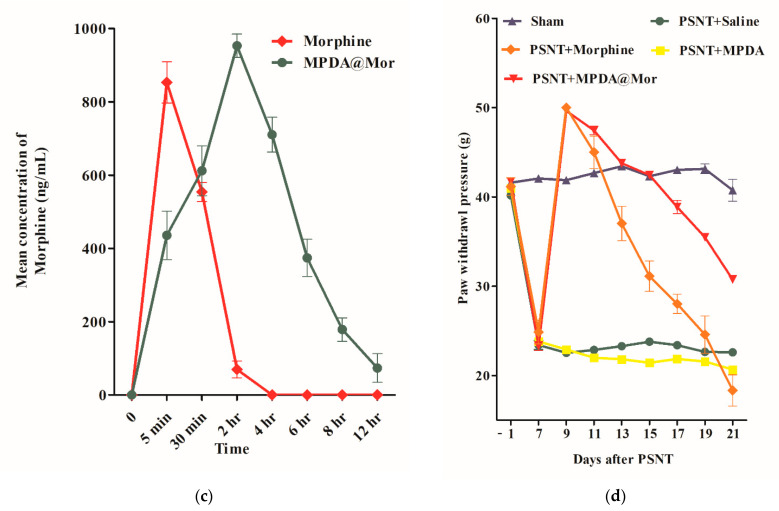
(**a**) Cumulative morphine release from MPDA@Mor in normal saline (pH 7.2). (**b**) Antinociceptive effect as a function of time after a single intraperitoneal injection of 15 mg/kg morphine and MPDA@Mor. The intensity of analgesia is expressed as % of maximal possible effect (MPE). (**c**) Pharmacokinetic profile of morphine and MPDA@Mor. (**d**) Effect of daily intraperitoneal (i.p) injection of either saline or morphine or MPDA@Mor on morphine antinociceptive tolerance (MAT) in sham or partial sciatic nerve transection rats. Differences between paw withdrawal threshold to tactile stimuli in rats with sham surgery (N = 6) and PSNT were assessed before nerve transection on the day of surgery (indicated as day −1 on the *x*-axis) and on day 7 after nerve transection. After PSNT, significant tactile sensitivity developed on day 7. The effect of MPDA, morphine, and MPDA@Mor on the mechanical paw withdrawal threshold was measured 60 min post i.p injection from days 9 to 21 after PSNT. *p* < 0.01.

The sustained-release of analgesics has been preferred over the short-acting analgesics for chronic pain management because of the longer duration of analgesia and reduced frequency of doses. Additionally, less frequent administration is shown to improve patient compliance with reduced toxic effects. Sustained analgesia and avoidance of sleep interruptions are other potential benefits [54]. The in vitro release of morphine from MPDA was studied in phosphate buffer saline at physiological pH (Figure 6a). Approximately 70% of morphine is released from the MPDA after 8 h in a sustained fashion without any initial burst release and is comparable with commercial morphine sustained-release formulations developed for human use [55].

The antinociceptive effects of morphine and MPDA@Mor were compared by administering a single intraperitoneal injection of 15 mg/kg morphine or MPDA@Mor (15 mg morphine equivalent) and the mechanical hypersensitivity to the paw withdrawal threshold was measured in neuropathic pain rats (Figure 6d). NP was initiated by PSNT. Before PSNT surgery, the mechanical hypersensitivity of the rat’s left paw was tested using a dynamic plantar aesthesiometer. Rats that passed a preset baseline (>35 g) were chosen for surgery. After surgery on the left leg, the mechanical hypersensitivity of the left paw was checked for 7 days. A reduction in the mechanical threshold was noticed in the nerve transected paw by day 7 (Figure 6d, green line). While sham animals did not display mechanical hypersensitivity (Figure 6d, violet line). The anti-allodynic effectiveness after a single injection of morphine and MPDA@Mor on day 7 of PSNT was compared by converting the data into % of MPE (Figure 6b). Morphine produced strong analgesia, peaking at 0.5 h post drug administration (Figure 6b, green line). The analgesic efficiency of morphine diminished with time; by the 3 h time point, there was a total loss of analgesic effect. These time-dependent analgesic effects of morphine result from the rapid metabolization and the decrease in plasma drug levels [56]. However, MPDA@Mor displayed a sustained analgesic action with a peak activity 2 h post drug injection and retained 50% analgesic efficacy 4 h post drug administration which could be attributed to the controlled release of morphine from MPDA.

To verify the sustained drug release properties of MPDA@Mor, a comparative pharmacokinetic study between morphine and MPDA@Mor samples was performed by analyzing morphine concentration in rat plasma. We determined pharmacokinetic parameters (Cmax, ng/mL) and t_1/2_ for morphine and MPDA@Mor in plasma. Cmax of morphine and MPDA@Mor were 853.2 and 953.2 ng/mL. MPDA@Mor had a significantly higher t_1/2_ (4.2 h) than free morphine (25 min). These results indicate that MPDA greatly improves the t_1/2_ of morphine with sustained analgesic effects.

MAT is a common side effect of chronic morphine use and is defined as a decline in antinociceptive effect with sustained administration of a constant dose. To check if morphine delivery through MPDA nanocarrier can minimize or delay MAT development, we have developed a MAT rat model by administering a dose of 15 mg/kg/i.p, daily, starting from day 9 of PSNT to day 21 in PSNT rats. Consistent with previous studies the morphine dose that was chosen resulted in MAT with a continuous decrease in mechanical paw withdrawal threshold from day 9 to 21 (Figure 6d, orange curve) [35,57]. On day 9 after injecting the first dose of morphine, the rats displayed a maximal analgesic effect to mechanical paw withdrawal threshold (50 g, i.e., cut-off threshold) whereas on day 21 the rats showed a threshold of 18 g which was less than that of PSNT saline groups (22.5 g) (Figure 6d, green curve). This hypersensitivity could be due to opioid-induced hyperalgesia. In comparison, morphine analgesic effects lasted till day 21 in MPDA@Mor-treated rats with a mean thermal withdrawal threshold of 30 g (Figure 6d, red curve). Though MPDA by itself did not have any significant analgesic effect at the doses we tested, our results show that MPDA@Mor can significantly delay the development of MAT (Figure 6d, yellow curve).

**Figure 7:**
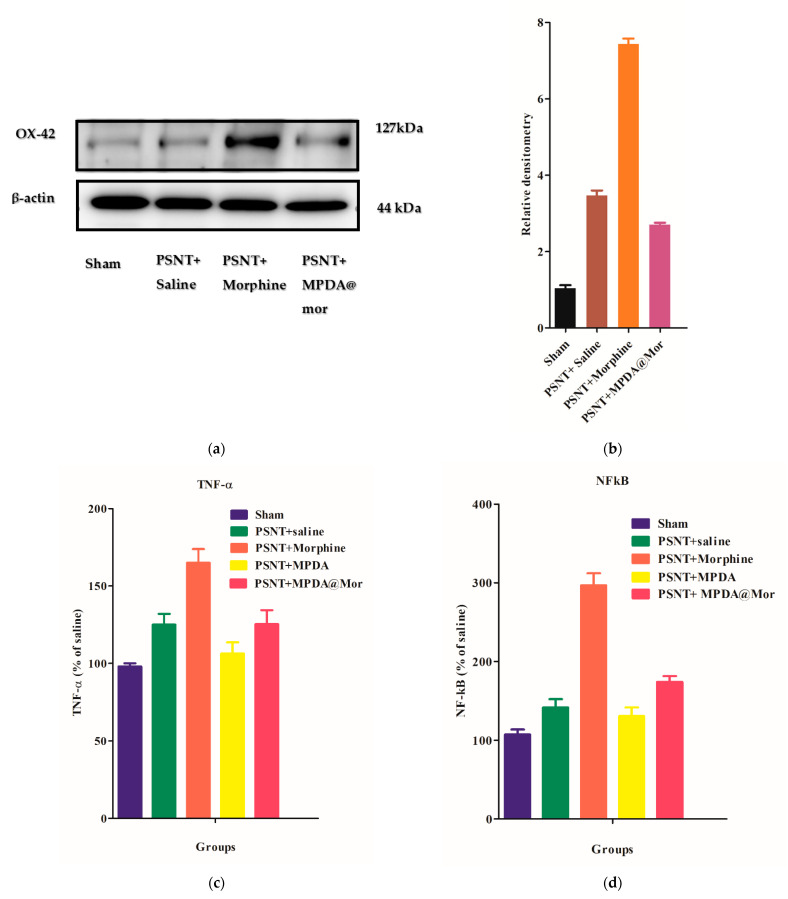
(**a**) Expression of OX-42 in the spinal cord dorsal horn of Sham, PSNT, morphine, and MPDA@Mor groups, immunoblotting of β-actin was used as a control (**b**) Quantification of OX-42 in spinal dorsal horn with Western blotting. Quantification of OX-42 was obtained with densitometric analysis and normalized with β-actin. (**c**) The effect of chronic administration of saline, morphine, MPDA, and MPDA@Mor on TNF-α in rat dorsal root ganglion (DRG). (**d**) The effect of chronic administration of saline, morphine, MPDA, and MPDA@Mor on NFkB in rat DRG.

An increasing amount of scientific evidence supports the role of spinal cord neuroinflammation in in the initiation and maintenance of NP and MAT [58,59]. Neuroinflammation contributes to NP and MAT by regulating the actions of immune cells, glial cell activation, and pro-inflammatory cytokine production in the central nervous system (CNS) [59]. Microglial cell activation is triggered in response to nerve injuries releasing proinflammatory cytokines, thus initiating the development of NP [60]. To delineate the underlying mechanisms by which MPDA@Mor can delay MAT, we first analyzed the glial cell activation. The OX-42 antibody is generally used to detect CD11b macrophages and is one of the most used markers for the detection of microglial activation in response to nerve injuries [61]. Experimental evidence shows that OX-42 expression levels are elevated on nerve injury [62]. Our results suggest that PSNT can activate microglial cells (Figure 7b, brown bar), and the activation is significantly increased upon chronic morphine administration (Figure 7b, orange bar) and that activation is significantly reduced in animals treated with MPDA@Mor, as demonstrated by Western blot analysis of the OX-42 protein.

Upon activation, microglial cells activate the M1 macrophages which act as the first line of defense offering innate immune responses through the activation of pro-inflammatory cytokines like TNF-α and activation of NFkb, along with the production of nitric oxide for inducible nitric oxide (iNOS) production [62,63,64]. It has been proven that targeting these pro-inflammatory cytokines can attenuate NP and the onset of MAT [65,66]. To investigate the effect of morphine and MPDA@Mor on pro-inflammatory cytokine levels and NFkb activation, the levels of these proteins were checked in rat dorsal root ganglion. Our results showed that the levels of TNF-α, and NFkb in the ipsilateral DRG increased significantly in the PSNT group (Figure 7c,d, green bars) when compared to sham control groups (Figure 7c, violet bars) due to the nerve damage [67]. Chronic infusion of morphine further elevated the levels of both proteins (Figure 7c,d, orange bars). Concurrently MPDA@Mor chronic administration significantly reduced TNF-α and NFkb protein levels in DRG compared with the morphine group (Figure 7c,d, red bars). Thus, we postulated that MPDA@Mor can alleviate MAT through the inhibitions of glial-derived pro-inflammatory cytokine activation which could be attributed to the antioxidant properties of MPDA, as the administration of exogenous antioxidants has been shown to attenuate MAT through the suppression of pro-inflammatory cytokine levels by several reports [7,8,9,10].

Apart from MAT, chronic administration of morphine is reported to cause significant damage to the hepatic antioxidant defense systems by the suppression of endogenous hepatic antioxidants and increase in the hepatic enzymes [68,69,70]. The liver is the primary site for the conversion of morphine and other opioids, and morphine metabolism is known to reduce the glutathione levels in the liver [71]. The suppression of hepatic antioxidants can cause oxidative injury to several tissues. Lipid peroxidation, DNA damage, protein oxidation, and apoptosis are some of the common effects associated with the suppression of endogenous antioxidant levels [72]. Some of the well-known substances that can restore the balance of morphine induced depletion of antioxidant enzymes are taurine, naloxone, N-acetylcystein and some plant-based natural antioxidants through attenuating morphine-induced oxidative stress [68,73,74,75]. To investigate the effect of morphine on hepatic endogenous antioxidant levels and lipid peroxidation, the levels of SOD, CAT, and MDA were tested in liver homogenates. It has been observed from our study that chronic morphine administration significantly reduces the hepatic antioxidant levels of SOD and CAT along with a significant elevation of malondialdehyde when compared to the control (Table 2). In contrast, treatment with MPDA@Mor significantly attenuated the suppression of hepatic SOD and CAT levels along with the reduction in lipid peroxidation as shown by MDA (Table 2), suggesting that delivery of morphine with an antioxidant nanocarrier can greatly reduce the hepatic oxidative stress induced by chronic morphine administration.

Additionally, liver enzyme analysis in serum revealed that chronic administration of morphine resulted in a significant increase in ALT, AST, and lactate dehydrogenase (LDH) enzymes compared with PSNT saline and sham groups (Table 3). Concurrently, chronic administration of MPDA@Mor stabilized the liver enzymes to a great extent. The increase in the activities of liver enzymes is a key indicator of liver cell impairment [76]. A single injection of morphine is known to increase dopamine and xanthine oxidation and subsequently increase ROS levels in rats [71]. Morphine is also known to metabolize free radicals and drastically enhances lipid peroxidation in MAT rats [70]. The antioxidant properties of MPDA have been shown to be responsible for its activity against various ROS related diseases, which could have protected the liver damage from morphine induced oxidative stress [23,24].

**Figure 8:**
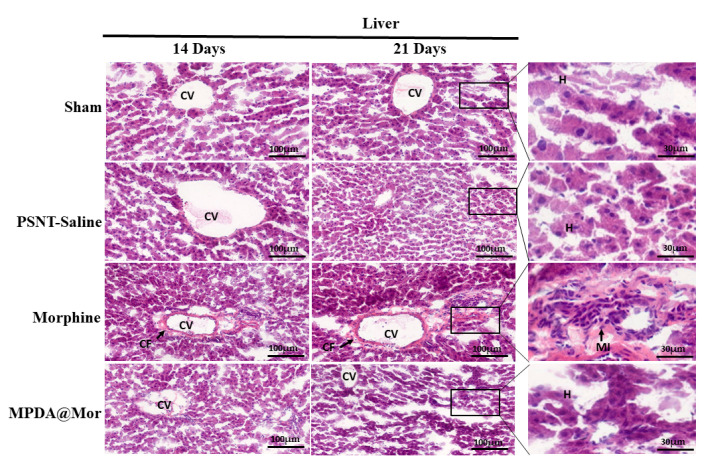
Hematoxylin and eosin stained cross-sections of the liver tissues of Sham, PSNT, morphine and MPDA@Mor groups, (Scale bar = 100 µM).

Considering the stimulatory effects of morphine on inflammatory enzymes and suppression of liver endogenous antioxidant enzyme activities we performed by hematoxylin and eosin staining (Figure 8) to observe liver histology as several reports confirmed the induction of liver toxicity on chronic administration of morphine [68,69,77]. Microscopically, the sham and PSNT saline rats display normal hepatocytes (H), and central vein (CV) morphology. The chronic morphine administration resulted in severe inflammation displaying liver fibrosis and monocyte infiltration (mi) and collagen fibers (cf) surrounding the central vein. Rats treated with MPDA@Mor presented an ameliorated condition.

Liver fibrosis is a body’s response to chronic liver injuries. It resembles wound healing with the involvement of inflammation, production of collagen fibers (CF) and non-collagenous extracellular matrix components, and tissue repair [78]. The present study proved that chronic morphine administration results in liver fibrosis as evident from the changes in hepatic pathology with an increase in collagen fiber production than control groups. Liver sections of MPDA@Mor did not show any significant differences from that of control and sham groups.

In summary, we developed an effective nano antioxidant platform for the elimination of morphine induced oxidative stress using MPDA-NPS as smart ROS scavengers. Our investigations with a series of experiments gave a comprehensive understanding about the underlying mechanisms of MPDA-NPS action. Spectroscopic results showed efficient antioxidative properties against ROS, showing their abilities in ROS scavenging. In vitro experiments demonstrated that MPDA-NPS can reduce the levels of ROS generation induced by morphine treatment. Using a partial sciatic nerve transection model of neuropathic pain, we further evidenced that i.p injection of MPDA@Mor can significantly delay MAT and limit hepatotoxicity by restoration of endogenous antioxidant levels in the liver and attenuation of liver enzyme upregulation induced by morphine. Moreover, the synthesized MPDA-NPS have a good biocompatibility with sustained analgesic effects. Finally, delivery of morphine with MPDA greatly attenuated microglial cell activation in the spinal dorsal horn, which can attenuate the aberrant hyperexcitability of spinal cord neurons to non-noxious and noxious stimulus. Collectively, our present study not only offer a comprehensive understanding of the oxidative stress alleviation properties of MPDA-NPs in neuropathic pain and possible mechanisms, but also would be highly advantageous in the development of an effective nano antioxidant platform for delivery of various cytotoxic pharmacological drugs.

## Figures and Tables

**Table 1:** The average zeta size, PDI, and zeta potential of samples before template extraction, after extraction, and drug loading.

Sample Name	Size (nm)	PDI	Zeta Potential (mV)
MPDA BE	141.71	0.211	−20.12
MPDA AE	149.63	0.179	−32.09
MPDA@Mor	190.13	0.091	−14.01

Note: PDI = Polydispersity index, BE = before template extraction, AE = after template extraction.

**Table 2:** Effects of morphine, MPDA@Mor on lipid peroxidation and antioxidant enzyme levels in Sham and PSNT rat livers.

Groups	MDA nmol/mg Protein	SOD (U/mg Protein)	CAT (k/g Tissue)
Sham	2.5 ± 0.51	14.23 ± 1.12	22.00 ± 1.89
PSNT + Saline	2.9 ± 0.71 *	12.6 ± 0.63 *	18.00 * ± 0.85 *
PSNT + Morphine	6.6 ± 0.61 **	8.5 ± 1.33 **	12.00 ± 1.30 **
PSNT + MPDA	3.1 ± 0.34	16.41 ± 1.92	26.00 ± 1.0
PSNT + MPDA@Mor	4.2 ± 0.41 *	10.32 ± 0.43 *	18 ± 2.51 *

* *p* ≤ 0.05, ** *p* ≤ 0.01.

**Table 3:** Effects of morphine, MPDA@Mor on liver enzyme functions in sham and PSNT rats.

Groups	ALT (U/L)	AST (U/L)	LDH (U/L)
Sham	30.12 ± 2.4	115 ± 7.00	510 ± 100.21
PSNT + Saline	35.6 ± 1.7 *	125 ± 10.0 *	620 ± 50 *
PSNT + Morphine	44.6 ± 2.3 *	240 ± 20.00 **	1500 ± 20 **
PSNT + MPDA	31.6 ± 1.4 *	108 ± 4.00	530 ± 98.61
PSNT + MPDA@Mor	34.9 ± 1.7 *	130 ± 18.00 *	850 ± 15 *

* *p* ≤ 0.05, ** *p* ≤ 0.01.

## Data Availability

The data presented in this study are available within the article.
